# Do Autistic Traits Predict Outcome of Cognitive Behavioral Therapy in Pediatric Obsessive-Compulsive Disorder?

**DOI:** 10.1007/s10802-023-01078-5

**Published:** 2023-05-18

**Authors:** Davíð R.M.A. Højgaard, Trine Wigh Arildskov, Gudmundur Skarphedinsson, Katja A. Hybel, Tord Ivarsson, Bernhard Weidle, Karin Melin, Nor Christian Torp, Per Hove Thomsen

**Affiliations:** 1 Department of Child and Adolescent Psychiatry, Aarhus University Hospital Psychiatry, Palle Juul-Jensens Boulevard 175, Entrance K, 8200 Aarhus N, Aarhus, Denmark; 2 Department of Clinical Medicine, Aarhus University, Aarhus, Denmark; 3 Faculty of Psychology, University of Iceland, Reykjavík, Iceland; 4 Regional Centre for Child and Youth Mental Health, Norwegian University of Science and Technology, Trondheim, Norway; 5 Department of Child and Adolescent Psychiatry, St. Olavs University Hospital, Trondheim, Norway; 6 Department of Child and Adolescent Psychiatry, Queen Silvia Children’s Hospital, Sahlgrenska University Hospital, Göteborg, Sweden; 7 Division of Mental Health and Addiction, Department of Child and Adolescent Psychiatry, Vestre Viken Hospital, Drammen, Norway; 8 Akershus University Hospital, Oslo, Norway

**Keywords:** Obsessive compulsive disorder, Cognitive behavioral therapy, Pediatric, Autistic traits

## Abstract

The first aim of this study was to explore whether children with obsessive compulsive disorder (OCD) and subclinical autistic traits can be differentiated from children with OCD without these traits based on clinical OCD-related characteristics, distinct OCD symptom patterns, and type of comorbidity. The second aim was to investigate whether autistic traits predict immediate and long-term outcome of exposure-based cognitive behavioral therapy (CBT) in pediatric OCD.

The participants in this study were a total of 257 children and adolescents aged 7–17 years, recruited from Denmark, Norway, and Sweden as a part of the Nordic long-term OCD treatment study (NordLOTS). Inclusion criteria were an OCD diagnosis based on DSM-IV criteria and a Children’s Yale-Brown Obsessive-Compulsive Scale (CY-BOCS) total severity score of 16 or higher. No children with a diagnosis on the autism spectrum were included. An Autism Spectrum Screening Questionnaire (ASSQ) cut-off score of ≥ 17 was used to define the group of OCD patients with autistic traits and all participants were treated with 14 weekly sessions of manualized CBT.

Comorbid attention-deficit/hyperactivity disorder and tic disorders, subclinical internalizing and externalizing symptoms, lower insight into OCD symptoms, more indecisiveness and pervasive slowness, and ordering/arranging OCD symptoms were found to be significantly associated with having OCD with autistic traits. No difference was found between the groups on treatment outcomes.

Results suggest that children and adolescents with OCD and autistic traits portray a different clinical profile than those without these traits, but that CBT is equally effective for those with and without autistic traits.

## Introduction

Obsessive-compulsive disorder (OCD) has been shown to affect between 0.5 and 3.0% of children and adolescents (Canals et al., 2012; Rapoport et al., 2000). OCD is characterized by the presence of recurrent and persistent obsessions in the form of intrusive and unwanted thoughts, urges, or images, along with compulsions, which are repetitive behaviors (including mental acts) to avoid, reduce, or escape those experiences (American Psychiatric Association, 2013). If left untreated, OCD can have severe consequences, including functional impairment (Skarphedinsson et al., 2015), reduced quality of life (Jensen et al., 2021; Weidle et al., 2015), and may pose an increased mortality risk in adults (Meier et al., 2016). Psychiatric comorbidity is seen in over two third of patients with OCD of all ages (Sharma et al., 2021). The psychiatric disorders most commonly co-occurring with OCD in children and adolescents are anxiety disorders, tic disorders, attention-deficit/hyperactivity disorder (ADHD), and depression (Ivarsson & Melin, 2008; Skriner et al., 2015; Torp, Dahl, Skarphedinsson, Thomsen, et al., 2015), with higher rates of ADHD found in children compared with adolescents (Smárason et al., 2021). Autism spectrum disorder (ASD) 1 is seen in around 1% of the general population (Zeidan et al., 2022), but the occurrence has been reported to be between 8 and 25% in clinical OCD samples (Griffiths et al., 2017a; Ivarsson & Melin, 2008; Martin et al., 2020; Rintala et al., 2017). Similarly, individuals diagnosed with OCD have been shown to have a nearly 4-fold increased likelihood of being diagnosed with ASD later in life (Meier et al., 2015). Subclinical autistic traits in pediatric OCD are also more frequent than in the general population, with a prevalence rate of up to 32.5% (Griffiths et al., 2017a; Ivarsson & Melin, 2008; Ozyurt & Besiroglu, 2017; Weidle et al., 2012). In an earlier study based on the sample of children and adolescents aged 7 − 17 with OCD used in the present study, the rate of autistic traits was found to be 10–17% depending on the scale cut-off chosen (Arildskov et al., 2016).Furthermore, the authors of a study on young children aged 5–8 years also found elevated autistic traits in their sample. They argued that this could be an artifact of measurement simply reflecting the shared topography of the disorders and/or imply a continuum of severity of autism features in those with OCD (Stewart et al., 2016).

### Clinical Characteristics, OCD Symptom Patterns, and Comorbidity in OCD with Autistic Traits

Population-based studies have shown that the rate of subclinical autistic traits is higher in boys than in girls (Constantino & Todd, 2003; Mattila et al., 2009; Posserud et al., 2006), whereas mixed findings have been reported in pediatric OCD samples. That is, some studies have reported no significant sex difference regarding subclinical autistic traits or co-occurring ASD in pediatric OCD (Ivarsson et al., 2008; Memis et al., 2019; Ozyurt & Besiroglu, 2017), while others have found a male preponderance among those with OCD and either subclinical autistic traits or ASD (Arildskov et al., 2016; Martin et al., 2020). Concerning OCD-related clinical correlates, findings regarding the association between autistic traits and OCD severity have been inconsistent: In the mentioned earlier study based on the sample of children and adolescents with OCD used in the present analysis, no significant relationship was found between social and communication difficulties scores and OCD severity, while the restricted, repetitive behavior score was found to be positively related to OCD severity (Arildskov et al., 2016). Another study found either significant or non-significant associations between OCD severity and subclinical autistic traits depending on the rating scale used to measure autistic traits (Stewart et al., 2016). A recent study found psychosocial functioning to be more impaired in young people with OCD + ASD compared with only OCD or ASD (Martin et al., 2020).

OCD symptom patterns are somewhat different in patients with OCD and co-occurring ASD. For instance, a study found that adults with both OCD and co-occurring ASD had more hoarding, symmetry, and repeating symptoms than those without ASD (Nakagawa et al., 2018). Hoarding has also been more common in children and adolescents on the autism spectrum with comorbid OCD, especially in those with greater social difficulties and in girls (la Buissonnière-Ariza et al., 2018). However, findings are inconsistent, and other studies have failed to find an association between hoarding symptoms and the presence of autistic traits or ASD in adults with OCD (e.g., (Anholt et al., 2010; Lewin et al., 2011)).

Children and adolescents with OCD and subclinical autistic traits have been shown to have a higher load of ADHD and tic disorders than those without autistic traits (Ivarsson et al., 2008). A similar pattern of comorbidity in children and adolescents with OCD and co-occurring ASD has also been found (Griffiths et al., 2017b; Lewin et al., 2011). Tic symptoms have previously been linked to a higher frequency of ADHD and higher levels of autistic traits in the sample used in this study (Højgaard et al., 2017). However, more studies are needed to confirm these findings.

### OCD with Autistic Traits and Cognitive Behavioral Therapy Outcomes

Several studies have evaluated cognitive behavioral therapy (CBT) in the treatment of OCD with co-occurring ASD. A review (which included 11 studies primarily based on pediatric samples) suggests that participants experienced an overall benefit from treatment as indicated by both clinician and parent-reported symptoms of OCD (Kose et al., 2018). One of the studies included in the review showed that those with OCD and co-occurring ASD had a substantially lesser decrease in OCD symptoms during treatment (38.3% vs. 48.2%) and lower remission rates at post-treatment (9.0% vs. 46.0%) when compared to participants with OCD without ASD (Murray et al., 2015). Another study investigated the effectiveness of CBT for child and adolescent OCD with either subclinical autistic traits or co-occurring ASD and found no group difference in treatment outcome (Wolters et al., 2016). Similarly, a study that followed OCD patients with and without co-occurring ASD for up to 11 years found no difference in the rate of improvement after CBT. However, they did find that the OCD + ASD group had higher OCD severity at follow-up (Nakagawa et al., 2018). A recent study found that youth with OCD + ASD were equally likely to receive CBT as those with OCD only, but that they were more likely to be prescribed medication as well as having an extended treatment course (Martin et al., 2020). At baseline and follow-up, those with OCD + ASD were more functionally impaired than those with OCD only, although significant improvement was seen after treatment. A randomized controlled trial has shown that functional behavior-based CBT is effective in treating obsessive-compulsive behaviors and improving quality of life in children and youths on the autism spectrum without intellectual disability (Vause et al., 2020). Moreover, preliminary findings suggest that intensive CBT is effective for the treatment of OCD in adolescents with co-occurring ASD (Iniesta-Sepúlveda et al., 2018). However, more studies are needed to investigate further the influence of subclinical autistic traits on CBT outcome in children and adolescents with OCD.

### Aims

The first aim was to explore whether children with OCD and subclinical autistic traits (OCD + AT) can be differentiated from children with OCD without these traits based on three different targets: (1) clinical OCD-related characteristics, (2) distinct OCD symptom patterns, and (3) type of comorbidity. The second aim was to investigate whether autistic traits predict immediate and long-term outcome of exposure-based CBT in pediatric OCD.

Based on a recent study (la Buissonnière-Ariza et al., 2018), we expected the OCD group with autistic traits to show more symptoms of hoarding compared with the OCD group without these traits. In line with findings from previous studies on OCD and co-occurring ASD (Griffiths et al., 2017b; Lewin et al., 2011), we expected a generally higher comorbidity load in the OCD + AT group compared with the OCD group. Finally, we expected subclinical autistic traits to predict a less favorable CBT outcome in pediatric OCD in line with previous findings (Kose et al., 2018; Nakagawa et al., 2018).

## Method

### Participants

The participants in this study were all included in the Nordic long-term OCD treatment study (NordLOTS), with a total of 269 children and adolescents aged 7–17 years, recruited from Denmark, Norway, and Sweden between September 2008 and June 2012 (Thomsen et al., 2013). Participants were referred from community health centers, general practitioners or by parents contacting the clinics directly. Inclusion criteria for the NordLOTS were an OCD diagnosis based on DSM-IV criteria confirmed with the Kiddie Schedule for Affective Disorders and Schizophrenia (K-SADS-PL), a Children’s Yale–Brown Obsessive–Compulsive Scale (CY-BOCS) total severity score ≥ 16, and no treatment with CBT six months prior to inclusion. Exclusion criteria were kept to a minimum, however, patients with ASD (except Pervasive Developmental Disorder-Not Otherwise Specified: PDD-NOS) or suffering from another psychiatric disorder with higher treatment priority than OCD (for example serious depression with suicidality or psychosis) were excluded. Patients with comorbid ADHD were allowed if treatment for ADHD had been stabilized at least three months prior to inclusion. Study rationale and inclusion procedures for the NordLOTS are described in detail elsewhere (Ivarsson et al., 2010; Torp, Dahl, Skarphedinsson, Thomsen, et al., 2015). Informed consent was obtained from all participants and their parents, and the trial was approved by the Danish, Norwegian and Swedish Committees for Medical and Health Research Ethics and the Medical Products Agencies.

In the present study, participants with co-occurring PDD-NOS (*n* = 1) and participants with missing or highly incomplete data on the Autism Spectrum Screening Questionnaire (*n* = 11) were excluded from the analyses. The final sample therefore consisted of 257 children and adolescents. Descriptive characteristics of this sample is shown in Table 1, more details have been reported elsewhere (Arildskov et al., 2016).

**Table 1:** Sample characteristics

	OCD + AT group (*n* = 25)	OCD group (*n* = 232)	*p-*value
Age, *M (SD)*^a^	12.16 (2.79)	12.86 (2.75)	0.227
Children/adolescents, *n*/*n*^b^	11/14	73/159	0.204
Boys/girls, *n*/*n*^b^	18/7*	107/125	0.014
ASSQ, *M(SD)*			
Total score	22.42 (7.89)	5.53 (4.34)	
Social difficulties	9.97 (3.48)	1.77 (2.12)	
Motor/tics/OCD	6.84 (3.80)	2.34 (1.97)	
Autistic style	5.60 (3.46)	1.41 (1.72)	

^a^ Independent *t*-test. ^b^ Pearson’s Chi-square test. *p < 0.05

Notes: *ASSQ* Autism Spectrum Screening Questionnaire, *AT* Autistic traits, OCD *Obsessive-compulsive disorder*

### Measures

#### Autism Spectrum Screening Questionnaire (ASSQ)

“The ASSQ is a questionnaire assessing features of ASD and consists of 27 items rated on a three-point scale (0 = no, 1 = somewhat, and 2 = yes), and includes statements such as “has a literal understanding of ambiguous and metaphorical language” and “is regarded as an “eccentric professor” by the other children” (Ehlers et al., 1999). The scale’s total score ranges from 0 to 54 and has an internal consistency of *α* = 0.88 (Arildskov et al., 2016). A three-factor structure of the ASSQ has been identified with a factor for autistic style, social difficulties, and motor/tics/OCD (Posserud et al., 2008). In the present sample, these factors have an internal consistency of *α* = 0.69, 0.82, and 0.72, respectively (Arildskov et al., 2016). The instrument has been proven a reliable and valid tool for screening in both clinical and general populations (Ehlers et al., 1999; Posserud et al., 2006, 2008). In the present study, the ASSQ was used as a measure of subclinical autistic traits and was completed at baseline by one or both parents/caregivers together. An ASSQ cut-off score of ≥ 17 for the total score(Posserud et al., 2009) was used to define the group of OCD patients with autistic traits. Moreover, the ASSQ total and subscale scores were used in the treatment outcome analyses.

#### Children’s Yale–Brown Obsessive–Compulsive Scale (CY-BOCS)

The CY-BOCS is a semi-structured interview used in the present study to evaluate OCD severity and symptom patterns (Scahill et al., 1997). The first part of the interview is a 74-item symptom checklist used to assess a broad range of current and past obsessions and compulsions. In this study, only current symptoms at baseline were used to investigate OCD symptom patterns. The second part is used to measure OCD severity and consists of 10 questions (five concerning obsessions and five concerning compulsions) rated on a five-point scale, with a total score ranging from 0 to 40 (Scahill et al., 1997). In addition, the CY-BOCS has six ancillary items covering insight, avoidance behavior, indecisiveness, pervasive slowness, pathological doubt, and overvalued responsibility (all rated on a five-point scale with a score range from 0 to 4). The CY-BOCS has been shown to be reliable and valid when used in samples of children and adolescents with OCD (Scahill et al., 1997; Storch et al., 2004). In the NordLOTS, the intra-class correlation coefficients (ICC) of inter-rater agreement for the OCD severity scale were as follows: obsessions ICC = 0.94 (95% confidence interval (CI): 0.85–0.97), compulsions ICC = 0.87 (95% CI: 0.67–0.93), and total score ICC = 0.92 (95% CI: 0.78–0.97) (Thomsen et al., 2013). Baseline CY-BOCS data were used to assess OCD-related clinical characteristics, whereas treatment outcome also included CY-BOCS ratings of OCD severity at post-treatment and at three follow-up time points (i.e., one, two-, and three-years follow-up).

#### The Clinical Global Impression Scales (CGI)

The Clinical Global Impression scales (Guy, 1976) are clinician ratings that measure severity and improvement. The CGI-Severity scale (CGI-S) was used by the clinician to rate the global severity of OCD symptoms. Ratings range from 0 (no illness) to 6 (extremely severe). It has been found to correlate strongly with the CY-BOCS total score in pediatric OCD patients and to be treatment sensitive (Busner & Targum, 2007). Baseline data were used in the present study as one measure of OCD-related clinical characteristics.

#### Kiddie Schedule for Affective Disorders and Schizophrenia –Present and Lifetime Version (K-SADS-PL)

The K-SADS-PL is a semi-structured diagnostic interview used to assess a broad range of child and adolescent mental disorders according to DSM-IV (Kaufman et al., 1997). The interview contains an introductory interview, a screening interview, and a diagnostic part. Items are scored as “not present,” “possible,” “in remission,” or “certain” (Kaufman et al., 1997). The K-SADS-PL has been shown to possess good interrater reliability (98%) and has a 1–5 week test–retest kappa of 0.80 for all contained anxiety diagnoses, including OCD (Kaufman et al., 1997). Convergent and divergent validity of the interview have also been shown to be good (Kragh et al., 2019; Lauth et al., 2010; Villabø et al., 2016). The interview was used in this study at baseline to assess OCD and comorbid disorders. Diagnoses were based on symptoms classified as certain only.

#### Child Behavior Checklist (CBCL)

The CBCL is used to evaluate child behavioral and emotional problems, as well as social competence (Achenbach & Rescorla, 2001). The scale has 113 items rated on a three-point scale (0 = not true, 1 = somewhat or sometimes true, and 2 = very or often true). CBCL has the following syndrome subscales covering internalizing and externalizing symptoms: anxious/depressed, withdrawn/depressed, somatic complaints, social problems, thought problems, attention problems, rule-breaking behavior, and aggressive behavior (Achenbach & Rescorla, 2001). In the NordLOTS sample, the internal consistency of the CBCL total score was *α* = 0.86 (Torp, Dahl, Skarphedinsson, Compton, et al., 2015). The CBCL was used as a measure of co-occuring internalizing and externalizing symptoms and was completed by the parent(s)/caregiver(s) at baseline.

### Treatment and follow-up

The first-step treatment included 14 weekly sessions (75 min) of exposure-based CBT. Individual therapy was given to the child in eight sessions for 45 min, while the parent was included for the remaining time, either alone or with the child. If needed, parents could also join for the whole session. In six sessions in between, both child and parents participated together. Non-responders to first-step CBT were randomized to either continued CBT or treatment with selective serotonin reuptake inhibitors. All participants were assessed for OCD severity and symptoms at six months, one year, two years, and three years after completing first-step CBT. Therapists in the study were child and adolescent psychiatrists, clinical psychologists, or certified psychotherapists with a minimum of five years of clinical experience. Assessments with CY-BOCS and K-SADS-PL were conducted by trained independent raters to ensure their reliability. Therapy adherence and fidelity were found to be good or very good. The treatment procedure has been described elsewhere (Thomsen et al., 2013; Torp, Dahl, Skarphedinsson, Thomsen, et al., 2015).

### Statistical Analyses

An ASSQ cut-off score ≥ 17(Posserud et al., 2009) and < 17 was used to define the groups of OCD patients with and without subclinical autistic traits. After applying this cut-off score, the OCD group with autistic traits (OCD + AT) consisted of 25 patients (9.7% of the total sample), while there were 232 OCD patients without autistic traits (Arildskov et al., 2016). Differences between these groups in terms of demographic variables were examined using independent *t*-test for continuous variables and Pearson’s *χ*2 test for categorical variables.

Exact logistic regression analyses with group status (OCD + AT or OCD) as the binary dependent variable were performed to investigate which variables (e.g., OCD severity, onset of OCD, CBCL syndrome scales) were related to and predictors of membership in the OCD + AT group while controlling for the influence of sex. Compared to regular logistic regression, exact logistic regression is a more reliable analysis when dealing with small and/or unbalanced sample sizes (Mehta & Patel, 1995), which is the case for the OCD + AT group (*n* = 25) and the OCD group (*n* = 232) in the present study.

Pearson’s *χ*2 tests or Fischer’s exact tests (when expected cell counts were *n* < 5) were used to compare frequencies of OCD symptom patterns and frequencies of total numbers of obsessions and compulsions between groups. The tic-related OCD subtype has been associated with pre-pubertal OCD onset, male preponderance (Højgaard et al., 2017; Leckman et al., 2010), and symmetry obsessions and ordering/arranging compulsions (Leckman et al., 2010), and although the literature is mixed in youths with OCD and tics (Conelea et al., 2015; Lewin et al., 2010) the potential confounding role of tic disorders needs to be taken into account. Hence, we performed exact logistic regression analyses controlling for the influence of tic disorders when significant results were obtained in analyses involving age of onset and OCD symptom patterns.

The initial treatment outcome analyses compared the baseline demographic and clinical characteristics between participants who responded (*n* = 177) and those who did not respond (*n* = 64) to initial CBT treatment (*n* = 16 were not available for assessment). We used *χ*2 tests for categorical variables and one-way analysis of variance (ANOVA) for continuous variables. The total sample of 257 participants was included in the analyses according to intent-to-treat (ITT) principles. Attrition to follow-up assessments was 37% at the two-year follow-up and increased to 38% at the three-year follow-up. Participants with missing and non-missing data were compared by baseline CY-BOCS total score, sex, age, and socioeconomic status. None of these comparisons showed significant differences, nor were differences found when estimating treatment outcomes. Therefore, we assumed that data were missing at random in the subsequent analyses.

To examine whether ASSQ predicted OCD severity after treatment and at follow-up (assessed using the CY-BOCS as a continuous measure) we used piecewise regression (Ryan & Porth, 2007; Singer & Willett, 2003). This model evaluates whether a shift in the outcome trajectory (CY-BOCS) occurs following the occurrence of a known event. In this study, the known event is the end of the first-step CBT. Piecewise regression was used to investigate the reduction in the CY-BOCS total score during first-step CBT compared with the three-year follow-up period. To address how a reduction could affect a participant’s outcome trajectory, we conducted a linear mixed-effects (LME) model that included two random effects (intercept, weeks since baseline, and week since end of step 1 CBT [time spline]) and fixed effects (ASSQ; interaction of time and ASSQ; and interaction of time spline and ASSQ). To this basic model, a series of discontinuous multilevel models for change were fitted to the data using restricted maximum likelihood estimation. The outcome modeled varied by the hypothesis under consideration and was evaluated by introducing to the “baseline” model a second level-1 individual growth trajectory with a discontinuity in both elevation and slope that marked the termination of the initial CBT treatment. The variable time spline, also a time-varying predictor, marked the passage of time following the start of follow-up. We conducted a series of LME analyses both using the continuous ASSQ total score and subscale scores, and the dichotomous ASSQ cut-off scores based on the participants above and below the 90th percentile on the ASSQ total score and subscales. These percentiles were estimated based on the current sample, and the following cut-offs were used: ASSQ total score ≥ 17, Autistic style ≥ 5, Social difficulties ≥ 8, and Motor/tics/OCD ≥ 7.

Exact logistic regression analyses were performed in Stata version 14 (StataCorp, 2015). Linear mixed effects/multilevel modeling was performed in SAS (SAS Institute, 2011). All other inferential and descriptive analyses were performed in IBM SPSS Statistics version 22 (IBM Corp, 2013). Tests were two-tailed, and a *p*-value of < 0.05 was considered significant. The study was explorative, and no correction of the alpha level was therefore applied.

## Results

The final sample comprised 257 children and adolescents with OCD without an ASD diagnosis. The OCD + AT group (*n* = 25) comprised 18 boys and seven girls, and the OCD group without AT (*n* = 232) comprised 107 boys and 125 girls. Sample characteristics and the ASSQ scores for the two groups are provided in Table 1.

When the OCD-related clinical correlates variables were examined, CY-BOCS total score (*p* = 0.035), compulsion severity score (*p* = 0.042), lower insight (*p* = 0.005), indecisiveness (*p* = 0.040) and pervasive slowness (*p* < 0.001) were found to predict having OCD + AT. The remaining CY-BOCS variables, CGI, and age of OCD onset were not found to be a significant predictor of having OCD with subclinical autistic traits. Scores, *p*-values, odds ratios, and 95% CI’s can be seen in Table 2.

**Table 2:** CY-BOCS scores as predictors of membership in the OCD + AT group while controlling for sex

	OCD + AT group (*n* = 25)	OCD group (*n* = 232)	OR	95% CI	Exact *p*-value^a^
CGI, *M (SD)*	3.64 (0.81)	3.42 (0.83)	1.48	0.85–2.63	0.152
CY-BOCS, *M (SD)*					
Total score	26.56 (4.58)*	24.50 (5.13)	1.09	1.00-1.19	0.035
Compulsion severity	13.36 (2.60)*	12.29 (2.67)	1.18	1.00-1.39	0.042
Obsession severity	13.20 (2.66)	12.21 (2.81)	1.16	0.99–1.36	0.064
Insight	1.68 (1.28)**	1.09 (0.93)	1.79	1.16–2.79	0.005
Avoidance	2.16 (1.07)	1.81 (1.05)	1.53	0.98–2.45	0.051
Indecisiveness	1.48 (1.26)*	1.00 (1.09)	1.46	1.00-2.13	0.040
Pervasive slowness	2.04 (1.06)***	1.05 (1.01)	2.42	1.54–3.95	< 0.001
Pathological doubt	1.08 (1.22)	0.96 (1.05)	1.14	0.76–1.69	0.547
Overvalued responsibility	0.48 (0.77)	0.87 (0.99)	0.65	0.35–1.11	0.135
Age of DSM OCD Onset, *M (SD)*^*b*^	10.43 (2.95)	11.69 (3.00)	0.88	0.75–1.01	0.067

^a^ For conditional score test. ^b^ Data available for n = 23 in OCD + AT group and n = 198 in OCD group. *p < 0.05, **p < 0.01, ***p < 0.001 (exact logistic regression). Notes:, *CGI* Clinical Global Impression scale, *CI* Confidence interval for Odds Ratio, *CY-BOCS* Children’s Yale-Brown Obsessive-Compulsive Scale, *DSM OCD Onset* Onset for fulfilling the diagnostic criteria for OCD, *OCD* Obsessive-compulsive disorder, *OCD + AT* OCD patients with autistic traits, *OR* Odds Ratio

**Fig. 1:**
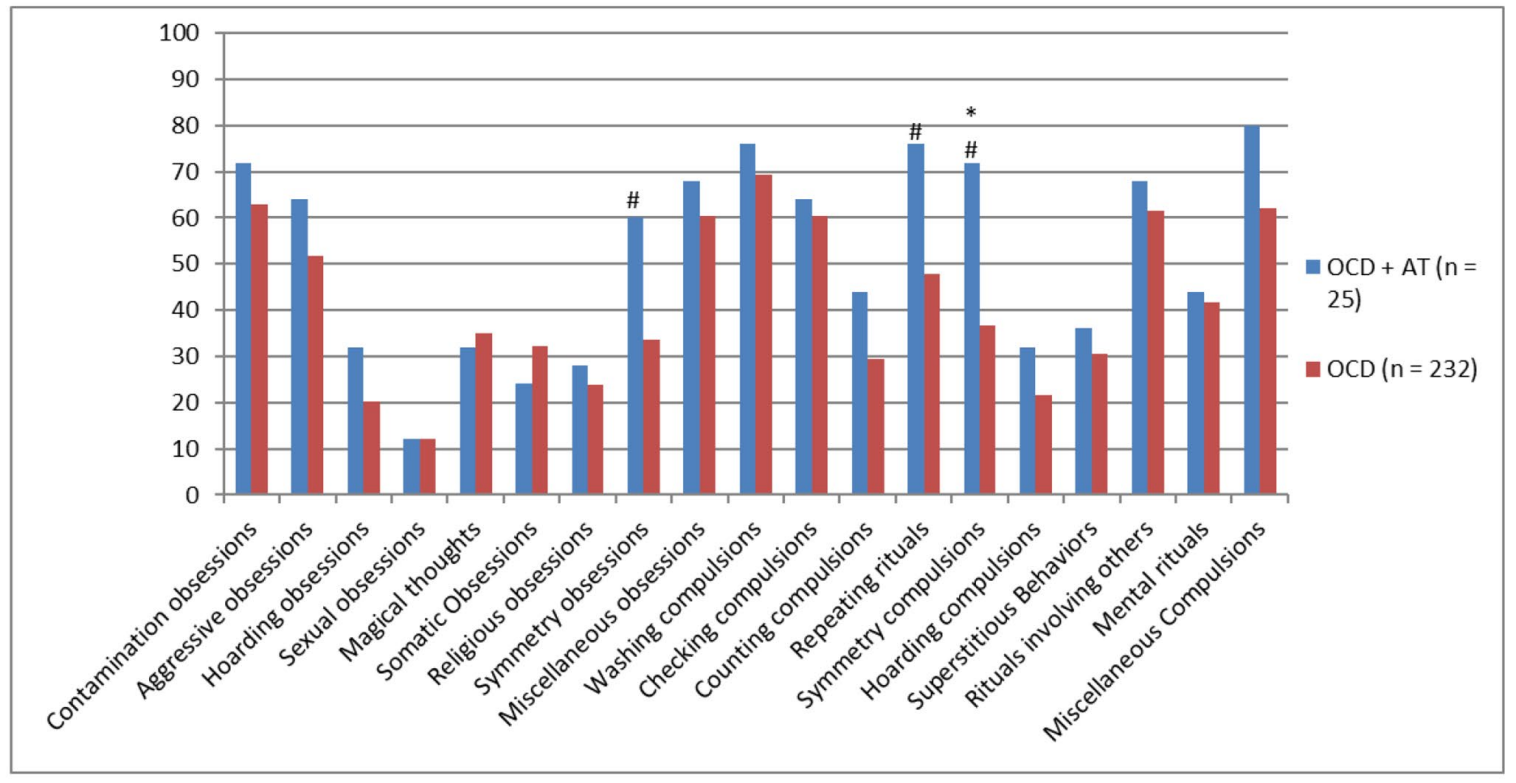
Percentage of participants with the different OCD symptom types in the OCD + AT group and the OCD group. (#*p* < 0.01 compared to the OCD group without controlling for tic disorders and sex (Chi-square tests). *p < 0.05 compared to the OCD group after controlling for tic disorders and gender (Exact logistic regression). Notes: *OCD* Obsessive-compulsive disorder, *AT Autistic traits*)

A comparison of the OCD symptom types between groups, based on the CY-BOCS categories, showed that symmetry obsessions (*p* < 0.01), repeating rituals (*p* < 0.01), and ordering/arranging compulsions (*p* < 0.01) were significantly more frequent in the OCD + AT group compared to the OCD group. Each OCD symptom according to the CY-BOCS checklist was rated as either present or not present. Figure 1 shows the percentage of participants in each group presenting with the different OCD symptom types defined using the CY-BOCS categories (e.g., contamination, symmetry, and hoarding obsessions). A comparison of the OCD symptom types between groups showed that symmetry obsessions (p < 0.01), repeating rituals (p < 0.01), and ordering/arranging compulsions (p < 0.01) were significantly more frequent in the OCD + AT group compared to the OCD group. However, only ordering/arranging compulsions remained significant (p < 0.05) after controlling for sex and the presence of tic disorders.

Results of the comorbidity analyses can be found in Table 3. The presence of comorbid ADHD and tic disorders were found to be a significant predictor of having OCD with subclinical autistic traits. In contrast, the presence of comorbid anxiety disorders, depressive disorders, and disruptive behavior disorders did not significantly increase the odds of having OCD with autistic traits. Higher scores on all CBCL syndrome scales, when analyzed with exact logistic regression (all *p*-values < 0.001), were found to predict having OCD + AT (Table 4).

**Table 3:** Comorbid disorders as predictors of membership in the OCD + AT group while controlling for sex

	OCD + AT group	OCD group	OR	95% CI	Exact *p-*value^a^
Anxiety disorders, *n (%)*	6 (24.0)	44 (19.1)	1.43	0.44–4.11	0.5848
Depressive disorders, *n (%)*	1 (4.0)	9 (3.9)	1.29	0.03–10.81	1.000
ADHD, *n (%)*	9 (36.0)***	13 (5.6)	7.06	2.13–23.56	< 0.001
Disruptive behavior disorders, *n (%)*	1 (4.0)	8 (3.5)	0.97	0.02–8.07	1.000
Tic disorders, *n (%)*	14 (56.0)***	32 (13.9)	7.11	2.70-19.19	< 0.001

^a^ For conditional score test. ***p < 0.001 (exact logistic regression). Comorbid disorders are based on n = 25 in the OCD + ASDS group and n = 231 in the OCD group. Notes: *ADHD* Attention/deficit-hyperactivity disorder, *CI* Confidence interval for Odds Ratio, *OCD* Obsessive-compulsive disorder, *OCD + AT* OCD patients with autistic traits, *OR* Odds Ratio

**Table 4:** CBCL syndrome scales as predictors of membership in the OCD + AT group while controlling for sex

		OCD + AT	OCD group		OR	95% CI	Exact *p*-value^a^
CBCL raw scores, *M(SD)*	**n** ^b^		**n** ^b^				
Anxious/Depressed	24	11.50 (5.32)	214	7.94 (4.73)	1.18	1.08–1.29	< 0.001
Withdrawn/Depressed	23	6.09 (2.99)	214	3.14 (2.70)	1.38	1.19–1.62	< 0.001
Somatic complaints	24	6.50 (4.01)	214	3.27 (3.05)	1.30	1.15–1.47	< 0.001
Social problems	23	7.38 (3.64)	213	2.28 (2.60)	1.50	1.30–1.76	< 0.001
Thought problems	23	11.04 (5.24)	213	6.84 (3.07)	1.32	1.17–1.50	< 0.001
Attention problems	23	10.09 (4.45)	211	4.17 (3.54)	1.37	1.22–1.56	< 0.001
Rule-breaking behavior	23	4.65 (2.97)	212	1.76 (1.99)	1.58	1.33–1.92	< 0.001
Aggressive behavior	23	15.07 (7.15)	213	5.92 (5.29)	1.22	1.14–1.32	< 0.001

^a^ For conditional score test. ^b^ Number of cases included in the analyses. *** *p* < 0.001 (exact logistic regression). Notes: *CBCL* Child Behavior Checklist, *CI* Confidence interval for Odds Ratio, *OCD* Obsessive-compulsive disorder, *OCD + AT* OCD patients with subclinical autistic traits, *OR* Odds Ratio

Regarding the treatment outcome, results from the LME analyses for ASSQ as continuous scores did not reveal any significant interaction between time (baseline to 3-year follow-up) and ASSQ scores, thereby showing that continuous ASSQ scores did not predict treatment outcome. For the dichotomous ASSQ variables (based on the 90th percentile, which represented a score of 17 or above for the ASSQ total score), the following between-group differences of estimates were significant: The ≥ 90th percentile groups based on the ASSQ total score, and social difficulties scale had significantly higher OCD symptom severity at baseline. However, no group differences in OCD severity were found at post-treatment or follow-up based on either the ASSQ total score or the social difficulties score. For the motor/OCD/tics subscale, the ≥ 90th percentile group had a significantly higher OCD severity score at post-treatment, although it was lower at 3-year follow-up. Estimated OCD severity scores (CY-BOCS) at baseline, post-treatment, 1-, 2-, and 3-years follow-up for both groups, along with group differences on CY-BOCS total scores, can be found in Table 5.

**Table 5:** Estimated baseline and post-treatment CY-BOCS total scores for ASSQ percentile groups

	CY-BOCS score in the ≥90th ASSQ percentile groups ^a^	CY-BOCS score in the < 90th ASSQ percentile groups ^b^	
	M (95% CI)	M (95% CI)	*t*-value (*p*-value)
**ASSQ total score** ^**c**^			
Baseline	26.7 (24.8, 28.7)	24.3 (23.7, 25.0)	2.29 (0.022)
Post-treatment	11.9 (9.1, 14.7)	10.0 (9.0, 11.0)	1.27 (0.204)
1-Year FU	9.0 (6.8, 11.2)	8.4 (7.6, 9.2)	0.49 (0.621)
2-Years FU	6.1 (3.9, 8.3)	6.8 (6.0, 7.6)	-0.61 (0.542)
3-Years FU	3.2 (0.5, 5.9)	5.2 (4.2, 6.2)	-1.39 (0.164)
**ASSQ Social difficulties**			
Baseline	26.7 (24.8, 28.7)	24.3 (23.7, 25.0)	2.27 (0.024)
Post-treatment	11.3 (8.5, 14.1)	10.1 (9.1, 11.1)	0.79 (0.433)
1-Year FU	8.6 (6.4, 10.8)	8.4 (7.7, 9.2)	0.15 (0.877)
2-Years FU	6.0 (3.8, 8.1)	6.8 (6.0, 7.5)	-0.71 (0.479)
3-Years FU	3.3 (0.7, 5.9)	5.1 (4.1, 6.1)	-1.28 (0.200)
**ASSQ Motor/tics/OCD**			
Baseline	25.8 (24.0, 27.6)	24.4 (23.7, 25.1)	1.39 (0.165)
Post-treatment	13.5 (11.0, 16.1)	9.7 (8.8, 10.7)	2.75 (0.006)
1-Year FU	9.8 (7.7, 11.8)	8.3 (7.5, 9.0)	1.34 (0.180)
2-Years FU	6.0 (3.9, 8.2)	6.8 (6.0, 7.6)	-0.66 (0.508)
3-Years FU	2.3 (-0.5, 5.0)	5.3 (4.3, 6.3)	-2.06 (0.040)
**ASSQ Autistic style**			
Baseline	25.4 (23.5, 27.2)	24.5 (23.8, 25.1)	0.86 (0.387)
Post-treatment	11.5 (8.8, 14.2)	10.0 (9.1, 11.0)	1.01 (0.315)
1-Year FU	8.8 (6.6, 11.0)	8.4 (7.6, 9.2)	0.34 (0.734)
2-Years FU	6.1 (3.9, 8.3)	6.8 (6.0, 7.5)	-0.57 (0.572)
3-Years FU	3.4 (0.6, 6.2)	5.1 (4.2, 6.1)	-1.16 (0.246)

Notes: *FU* Follow-up, *CI* Confidence interval, *ASSQ* Autism Spectrum Screening Questionnaire, *OCD* Obsessive-compulsive disorder, *CY-BOCS* Children’s Yale-Brown Obsessive-Compulsive Scale

## Discussion

The main results from the study were that the group consisting of 25 children with OCD and subclinical autistic traits presented with a different clinical profile compared to those without these traits, whereas CBT was equally effective for those with and without autistic traits. The proportion of the sample displaying autistic traits as assessed with the ASSQ is similar to the one found in Ivarsson and Melin (2008), but lower than found in Griffiths et al. (2017a; Stewart et al. (2016)) found higher autistic trait scores when using the Social Responsiveness Scale (SRS) but not when using the Social Communication Questionnaire (SCQ). This difference may therefore at least partially be explained by the different measures and cut-off scores used.

Of the different OCD-related clinical characteristics measured by the six ancillary CY-BOCS items characterizing the OCD + AT group, pervasive slowness was found to have the highest odds ratio. As pervasive slowness covers disturbance of inertia related to difficulties initiating and completing tasks (Scahill et al., 1997), this finding may be explained by the high rate of comorbid ADHD (36.0%) found in the OCD + AT group. In addition, difficulties initiating and completing tasks is an overlapping feature of ADHD and ASD. Poorer insight and greater degree of indecisiveness were also found to increase the odds of having OCD with autistic traits, although to a lesser extent than pervasive slowness. With regard to insight into the exaggerated nature of OCD symptoms, Ivarsson et. al(2008) did not find lack of insight to be correlated with scores on the ASSQ, seemingly contradicting our results. However, we used a categorical ASSQ cut-off score to define two groups (one with subclinical autistic traits and one without autistic traits) and investigated differences between these groups, while Ivarsson et al. (2008) used a continuous ASSQ score which may contribute to the difference in results. Nevertheless, poor insight is common in individuals on the autism spectrum (Wikramanayake et al., 2018), and another study reported that a higher proportion of adults with OCD and subclinical autistic traits showed poorer insight compared to adults with OCD alone (Mito et al., 2014), which is in line with our findings. Indecisiveness has to our knowledge not been examined in this group of patients previously, however, decision making has been found to be slower in adults on the autism spectrum, indicating an inclination to collect more information before making a decision, hence making it more difficult (Luke et al., 2012; Vella et al., 2018). Further studies are needed to investigate the nature of insight, indecisiveness, and pervasive slowness in people with concurrent OCD and subclinical autistic traits.

The group with OCD and subclinical autistic traitswas found to have significantly higher frequency of symmetry obsessions, repeating rituals, and ordering/arranging compulsions compared to the group without these traits, but not hoarding obsessions and compulsions as we expected. Only the frequency of ordering/arranging compulsions remained to differ significantly between groups after adjusting for tic disorders. These results are consistent with the commonly seen clinical picture of children and adolescents on the autism spectrum where symmetry, ordering and repetition are described (Nakagawa et al., 2018). It is also possible that the CY-BOCS rating may be capturing these symptoms as symptoms of OCD, while they may in fact be more associated with the subclinical autistic traits in this group of OCD patients, with especially repetitive compulsions being topographically similar between OCD and the autism spectrum (Jiujias et al., 2017).

Concerning comorbidity, we found that 36.0% of those with OCD and autistic traits had comorbid ADHD compared to only 5.6% in the OCD group without autistic traits, which is in line with previous research (Anholt et al., 2010; Ivarsson et al., 2008) and consistent with our hypothesis. Similarly, the OCD + AT group also scored higher on related CBCL subscales (attention problems, rule-breaking behavior, and aggressive behavior). As expected, we found the OCD + AT group to have a considerably higher frequency of tic disorders than the OCD group without autistic traits (56.0% vs. 13.9% respectively). Although we did not find a difference between the groups regarding anxiety disorders and depressive disorders, the OCD + AT group was found to score higher on the anxious/depressed, somatic complaints, and withdrawn/depressed syndrome scales of the CBCL. A possible explanation for this is that the CBCL syndrome scales could be measuring elements overlapping between the disorders or possibly that the OCD + AT group is characterized by a higher load of subclinical psychopathology in general as they had higher scores on all CBCL syndrome scales. Given the high co-occurrence of ADHD (36%) and tic disorders (56%) in the group with OCD and autistic traits, we cannot rule out that this group may be better described as having neurodevelopmental features in general. Future studies are recommended to examine this in broader samples. Another explanation may be that our sample included few patients with depressive disorders and disruptive behavior disorders, and the analyses may therefore have failed to find group differences.

The pre-treatment OCD severity was found to be higher by an average of 2.05 points in the OCD + AT group, but previous studies have shown conflicting results regarding this (Arildskov et al., 2016; Stewart et al., 2016). The difference was only significant concerning compulsions but not obsessions, and although significant, this modest difference is not likely to be clinically meaningful. Both groups of patients improved significantly during the course of CBT and the level of improvement was not different between groups. This indicates that both the immediate and long-term effectiveness of CBT for child and adolescent OCD is not impacted by the presence of subclinical autistic traits. These results conflict with our hypothesis as we expected a less favorable outcome for the group with OCD + AT. The reason for this may be that most previous research was based on OCD with co-occurring ASD and not subclinical autistic traits (Kose et al., 2018). The only study in the 2018 review, which included OCD patients with both diagnosed ASD and subclinical autistic traits found no effect on treatment outcome (Wolters et al., 2016). This may indicate that when autistic traits are on a subclinical level, they do not interfere with CBT outcome of OCD.

The strengths of this study include a large sample size and the use of semi-structured standardized interviews in the diagnostic assessment of OCD and the use of a validated questionnaire (Ehlers et al., 1999) to assess autistic traits. The limitations of our study were that the assessment of autistic traits was based on parents rating of the ASSQ only, increasing the risk of erroneously rating OCD symptoms as autistic traits. Furthermore, ASSQ may not be an ideal scale to evaluate autistic traits as it includes items that are not core features of the autism spectrum as well as items that could be confused with OCD (e.g., “has difficulties in completing simple daily activities because of compulsory repetition of certain actions or thoughts”; Ehlers et al., 1999). Future studies are therefore recommended to investigate the potential overlap between the CY-BOCS and the ASSQ or similar rating scales assessing autistic traits. Also, the K-SADS was used to obtain comorbidity diagnoses, including co-occurring ASD. However, it has been shown to be less than optimal regarding ASD diagnoses when used alone (Jarbin et al., 2017). It is therefore possible that the instrument may have missed ASD diagnoses according to DSM-IV criteria in both groups in our sample. Due to the exclusion of children and adolescents on the autism spectrum in the present study, we were not able to determine the usefulness of the specific ASSQ cut-off in diagnosing or screening for ASD in a sample of children and adolescents with OCD. Furthermore, our sample also had relatively low comorbidity rates compared to other pediatric OCD studies (Sharma et al., 2021). Finally, OCD diagnoses were not reassessed post-treatment, limiting outcome evaluation to a CY-BOCS severity score.

## Conclusion

Results from the study suggest that children and adolescents with OCD and subclinical autistic traits present with a different clinical profile than those without these traits, displayed by a higher frequency of comorbid ADHD and tic disorders, lower insight, more indecisiveness, and increased pervasive slowness. This group also showed a different OCD symptom pattern with more obsessions related to symmetry and compulsions related to repetition and ordering/arranging. However, an encouraging and important finding for clinicians was that the presence of subclinical autistic traits did not impact the outcome of manualized CBT for OCD.

## Footnotes

1 For clarity, we use the terms “autism spectrum disorder” (ASD) or “on the autism spectrum” when referring to the diagnosis (e.g., in the Diagnostic and Statistical Manual of Mental Disorders) or studies including individuals with both OCD and co-occurring autism spectrum conditions. In contrast, we use the term “autistic traits” (which is more in alignment with identity-first discourses; e.g., (Bottema-Beutel et al., 2021) to refer to (subclinical) autistic characteristics in the population (typically assessed using rating scales) that do not warrant a diagnosis.
